# Glucose monitoring from gingival crevicular fluid blood among chronic periodontitis patients at Ar Rass, Saudi Arabia

**DOI:** 10.6026/973206300200337

**Published:** 2024-04-30

**Authors:** Faris Saleh A Alqazlan, Saad Obaid Alazmi, Nubesh Khan Syed, Abdulaziz Abdulrhman A Alshumaym, Abdullah Ahmad A Aloyouni, Khalid Abdullah G Alfuryah, Saleh Suliman S Almuzaini

**Affiliations:** 1 Department of Periodontology and Implant Dentistry, College of Dentistry, Qassim University, Saudi Arabia

**Keywords:** Chronic Periodontitis, Gingival crevicular fluid blood, Diabetes mellitus, Fingerstick blood glucose

## Abstract

Gingival crevicular fluid blood (GCFB), during periodontal probing is useful to assess blood sugar levels using a glucometer. Hence, blood glucose levels in chronic periodontitis with and without diabetes were measured using gingival crevicular fluid and compared to finger stick blood glucose levels (FSBG). A total of 48 patients (24 diabetics and 24 non-diabetics) with chronic periodontitis who matched the inclusion criteria were divided into two groups, Group I and Group II, respectively. The entire patient's plaque and Russel's periodontal indices were recorded and a glucometer was used to measure random blood glucose from the gingival crevicular fluid and finger pricks. A positive association between the blood glucose level measured by a fingerstick and the gingival crevicular fluid is observed. Thus, GCFB can be used as a reliable chairside diagnostic technique for diagnosis diabetes in a dental setting.

## Background:

To stay healthy and prevent conditions from becoming worse, health indicators must be periodically evaluated. Regular examinations of blood are a method to do this. Patients can become reluctant to inquire about a medical examination as they're uninformed that a dentist may require a medical consultation, which is essential prior performing any dental treatments [1]. Dentists are in an excellent place to help patients diagnose pre-diabetes early and improve public awareness of diabetes mellitus. It has become widely recognized that diabetes mellitus and periodontitis have a relationship in both directions, with the presence of one disorder making the other progressively worse [2]. Dental health care providers are expected to meet a high number of patients with a combination of diabetes mellitus and periodontitis owing to an intimate link that exists between the two conditions. Therefore, screening for bodily issues that could affect a patient's dental treatment or general health is the dentist's job. One of the most suitable occasions for screening for diabetes is during a dental appointment, especially for high-risk groups [3]. In recent years, diabetes patients have become more likely to check their blood glucose levels at home utilizing glucometers. Glucometers are effortless to use, inexpensive, as well as precise enough [4]. The glucometer has the potential to be an effective instrument at the dental office for evaluating patients who might be suffering from diabetes and perhaps minimizing the possibility of serious complications [5]. When the gingival sulcus becomes inflamed, blood, referred to as gingival crevicular blood, can drain from the sulcus during a periodontal examination. Gingival crevicular blood, which is obtained from sites with inflamed gingival tissue and bleeding upon probing, is less invasive and traumatic than using a sharp lancet to puncture the finger to obtain capillary blood for blood glucose measurement [6]. Upon probing, the gingival crevicular blood volume must be sufficient, and the blood must be free of contamination from contact with the gingiva and teeth [7]. Accurate self-monitoring devices have recently been developed to test small quantities of blood (less than 2 µl), and their accuracy has shown to be satisfactory [8]. Therefore, it is of interest to monitor glucose in gingival crevicular fluid blood among chronic periodontitis patients in Saudi Arabia.

## Materials and Methods:

The present cross-sectional research was done on an unknown people of outpatients who reported to the Dental Clinics, College of Dentistry, and Qassim University, Saudi Arabia. Before the collection of the sample, the study was approved by the Committee of Research Ethics, Deanship of Scientific Research, Qassim University (23-42-10) and it was conducted according to the Helsinki Declaration. After estimating the sample size, it was fixed as 48 and the participants were categorized into 2 groups (Group I-24Chronic Periodontitis patients with diabetes mellitus and Group II-24 non-diabetes Chronic Periodontitis patients).After obtaining written informed consent from each patient, detailed case history and clinical examination were taken along with a plaque index and Russell's periodontal index. Based on the patient's history and medical records, diabetic and non-diabetic patients were selected for the study. The American Academy of Periodontology's suggested clinical and radiological criteria for the new classification scheme for periodontal and peri-implant diseases and disorders were used to diagnose individuals with periodontitis [9]. Patients with chronic periodontitis, both male and female, aged 30 to 60 years, with or without diabetes mellitus were incorporated in this research. Patients taking anticoagulant drugs, non-steroidal anti-inflammatory drugs, vitamin C supplements, any conditions requiring antibiotic prophylaxis, pregnancy, teeth with suppuration, and systemic diseases other than diabetes were excluded from the study.

The Accu-check Glucometer, a commercially available glucometer, was used in this study. The glucose in the blood sample is converted to gluconolactone by the strip's glucose dehydrogenase. Two electrons are released from this reaction and react with an electron acceptor in a coenzyme (PQQ). A safe electrical current is produced by the entire reaction, which the metre interprets as blood glucose.

## Sample collection:

## Gingival crevicular fluid blood:

The Gingival crevicular fluid blood was drawn from patients in both the groups in order to evaluate the random blood glucose levels. As a way minimizes contamination, patients were instructed to rinse their mouths with mouthwash comprising 0.2% chlorhexidine. Maxillary anterior teeth were picked for glucose readings as they provide the most favorable access for collecting gingival crevicular blood. The test site was chosen based on the location of the most evident visual changes in inflammation. Cotton rolls were utilized to isolate that area, and air drying prevented saliva contamination. Bleeding on probing was noted during routine periodontal examination using William's periodontal probe. This method has been suggested as a noninvasive way to draw blood samples. The blood seeping from the gingival border was then brought into touch with the upper edge of a test strip that had been preloaded into a glucometer, preventing contact with the gingiva or teeth. By means of capillary action, 3µl of blood are automatically pulled into the strip until the conformation window is entirely filled. The glucose measurements obtained from the glucometer screen are then recorded in mg/dL.

## Finger-prick blood:

The samples for the fingerstick blood were ideally drawn from the soft tissue surface of the patient's index finger on the less dominant hand. After wiping the soft tissue surface with the surgical spirit, the finger was pricked with a sterile lancet, and a drop of blood was allowed to develop on the finger. After eliminating the first drop of blood, the test end of the strip was placed to the bleeding site and held there until the gadget buzzed and exhibited the random blood glucose readings in mg/dL on the screen.

## Statistical analysis:

A software application (SPSS Version 22 Software, IBM Statistics, and USA) was used to evaluate the collected data. The clinical parameters of the two groups were compared using the Mann Whitney Test. The significance of variations in blood glucose levels between the diabetic and non-diabetic groups analyzed by finger prick and gingival crevicular blood was assessed using an independent-t test. P values less than 0.05 were regarded as significant.

## Results:

The study had 48 chronic periodontitis patients in total, of whom 32 (66.66%) were males and 16 (33.33%) were females. The patients were allocated into two groups: Group I was composed of 24 patients with diabetes mellitus and Group II consisted of 24 non-diabetes patients. The participants' ages ranged from 30 to 60 years old, with the mean age of group I was45.89 and group II being 45.76 (Table 1). There isn't a noticeable statistically significant difference in the mean age between the two groups. The mean plaque index and mean Russel's periodontal index for the two groups were 25.79 and 23.21respectively, and the p value for none of the clinical characteristics was significant (Table 2). Patients belonging to group I and II had GCFB blood sugar values of 143.04 mg/dl and 110 mg/dl, respectively. The mean result for FSBG was 156.50 mg/dl in patients in group I and 108.91 mg/dl in individuals in group II. Table 3 and Figure 1 (see PDF) shows the extremely significant P value of 0.001 for the comparison of GCFB glucose and FSBG in both groups.

## Discussion:

People with diabetes mellitus have severe gingival bleeding with more aggressive periodontal destruction [10- 11,12]. The fact that diabetes and periodontitis are correlated, the dentist office could represent a largely unexplored potential for diabetes screening. Dentists, especially periodontists, are likely to come through undetected diabetic patients in their routine dental practice. Consequently, diabetes screening aids in preventing the long-term problems that lead to the high morbidity and death rates of diabetic individuals receiving periodontal therapy [13]. Kassim *et al.* postulated that while most dentists are interested in implementing medical screening in their practices, unfortunately many are not familiar with the various screening techniques [14]. Dental professionals are interested in checking the blood glucose levels using glucometers, since it is easy to use, reasonably priced, and accurate enough to be effective [15]. In the current research, the glucose readings from the patient's GCFB were compared to those from a conventional finger stick sample. Considering the OneTouch Select Simple Glucometer only requires 2 µl of blood to analyze the blood sample, it was employed in our investigation. In the study by Parihar *et al.* gingival index and probing depth were demonstrated to be significant periodontal indicators [16]. Similarly, both groups in the current study applied the periodontal index and the plaque index as indicators.

In both diabetic and non-diabetic patients, a random blood glucose level was recorded in finger prick and gingival crevicular blood. We observed a statistically significant link between GCFB and FSBG. Contrary to the current investigation, Rapone *et al.* [17] and Debnath *et al.* [18] revealed a negative connection between capillary blood glucose and gingival crevicular blood. The current study's findings correlate with those of investigations performed by Müller *et al.* [8] and Parker *et al.* [19]. In the current research, there is a strong interrelationship between these two measurements in patients with substantial bleeding upon probing. This demonstrates that, in agreement with other research, GCFB may be used in dentistry offices to screen for diabetes. With regard to the outcomes and restrictions of the current investigation, the goal was to establish that gingival crevicular fluid blood can be utilized as chairside diabetes investigations utilizing a glucometer. The patients did not experience any sensation of pain throughout the periodontal probing and GCFB blood collection process. During the probing or sample collection, the patients did not express any sensation of discomfort. In addition to gathering the information required for the diagnosis of periodontal disease, a GCFB sample can be used for diabetes screening. Thus, the bleeding blood sample that would typically be discarded away is used for evaluating glucose levels. Despite the fact that this is not a confirmatory test for diabetes, the people who are suspected should have additional testing done. This study has certain shortcomings as well. Since venous blood is the gold standard for measuring glucose levels, we did not draw any of it. Additionally, the subjects underwent only a diabetic screening; a confirmatory test was not performed following the screening. This experiment has a small sample size. Therefore, larger sample sizes and more precise techniques for measuring and identifying GCFB glucose should be included in future study in order to achieve the goal of early diabetes mellitus detection.

## Conclusion:

The correlation between GCFB glucose and FSBG is shown. Hence, GCFB is a suitable chair-side screening during routine periodontal examinations in dental clinics. The method outlined is safe, simple to use, repeatable, comfortable for the patient, economical and may thus contribute to a rise in the number of diabetes screenings conducted in dental clinics.

## Figures and Tables

**Table 1:** Gender and age allocation

	**Gender**					**Age**
Group	Male	%	Female	%	Total	Mean Age
Group I	15	62.50%	9	37.5	24	45.89
Group II	17	88.23	7	29.16	24	45.76

**Table 2:** Comparing clinical parameters between groups

		**N**	**Mean Rank**	**p-value**
Plaque Index	Group I	24	25.79	0.497
	Group II	24	23.21	
Russel's Index	Group I	24	24.92	0.83
	Group II	24	24.08	
P-value based on Mann Whitney Test; * = Statistically Significant (p < 0.05)

**Table 3:** Intergroup Comparison of Finger-Stick Blood Glucose and Gingival Crevicular Fluid Blood

		**N**	**Mean**	**Standard Deviation**	**p-value**
Gingival Crevicular Fluid Blood glucose	Group I	24	143.04	49.21	0.006*
	Group II	24	110	26.05	
Finger-Stick Blood glucose	Group I	24	156.5	48.78	< 0.001*
	Group II	24	108.91	26.47	
P-value based on Independent-t-Test *=Statistically Significant (p < 0.05)
